# Apomorphine is a potent inhibitor of ferroptosis independent of dopaminergic receptors

**DOI:** 10.1038/s41598-024-55293-1

**Published:** 2024-02-27

**Authors:** Akihiko Miyauchi, Chika Watanabe, Naoya Yamada, Eriko F. Jimbo, Mizuki Kobayashi, Natsumi Ohishi, Atsuko Nagayoshi, Shiho Aoki, Yoshihito Kishita, Akira Ohtake, Nobuhiko Ohno, Masafumi Takahashi, Takanori Yamagata, Hitoshi Osaka

**Affiliations:** 1 Department of Pediatrics, Jichi Medical University, Shimotsuke, Japan; 2 Division of Inflammation Research, Center for Molecular Medicine, Jichi Medical University, Shimotsuke, Japan; 3 Diagnostics and Therapeutics of Intractable Diseases, Intractable Disease Research Center, Graduate School of Medicine, Juntendo University, Tokyo, Japan; 4 Department of Life Science, Faculty of Science and Engineering, Kindai University, Osaka, Japan; 5 Department of Clinical Genomics & Pediatrics (Faculty of Medicine), Saitama Medical University, Saitama, Japan; 6 Center for Intractable Diseases, Saitama Medical University Hospital, Saitama, Japan; 7 Department of Anatomy, Division of Histology and Cell Biology, School of Medicine, Jichi Medical University, Shimotsuke, Japan; 8 Division of Ultrastructural Research, National Institute for Physiological Sciences, Okazaki, Japan

**Keywords:** Drug development, Clinical pharmacology, Drug delivery, Pharmaceutics

## Abstract

Originally, apomorphine was a broad-spectrum dopamine agonist with an affinity for all subtypes of the Dopamine D1 receptor to the D5 receptor. We previously identified apomorphine as a potential therapeutic agent for mitochondrial diseases by screening a chemical library of fibroblasts from patients with mitochondrial diseases. In this study, we showed that apomorphine prevented ferroptosis in fibroblasts from various types of mitochondrial diseases as well as in normal controls. Well-known biomarkers of ferroptosis include protein markers such as prostaglandin endoperoxide synthase 2 (PTGS2), a key gene for ferroptosis-related inflammation PTGS2, lipid peroxidation, and reactive oxygen species. Our findings that apomorphine induced significant downregulation of PTSG2 and suppressed lipid peroxide to the same extent as other inhibitors of ferroptosis also indicate that apomorphine suppresses ferroptosis. To our knowledge, this is the first study to report that the anti-ferroptosis effect of apomorphine is not related to dopamine receptor agonist action and that apomorphine is a potent inhibitor of ferroptotic cell death independent of dopaminergic receptors.

## Introduction

Mitochondrial disease is a genetic disorder consisting of various clinical phenotypes, including Leigh syndrome (LS), myopathy encephalopathy lactic acidosis, stroke-like episodes (MELAS), Kearns-Sayre syndrome (KSS), and mitochondrial cardiomyopathy^1,2^. At present, no curative treatment is available and effective treatment options are eagerly awaited. In our previous study, we screened a chemical library based on drugs already approved for central nervous system (CNS) diseases using fibroblasts from two patients with LS and two patients with MELAS using an L-buthionine-S, R-sulfoximine (BSO)-induced cell viability assay. BSO has been used to induce cell death by inhibiting glutathione (GSH) biosynthesis, which leads to the overproduction of reactive oxygen species (ROS)^3^. In addition, BSO has been recently reported to trigger ferroptosis in various cancer cells^4,5^.

While searching for potential therapeutic drugs for mitochondrial diseases, we identified apomorphine as a potential therapeutic drug for LS and MELAS^6^. Apomorphine is a known treatment for Parkinson’s disease (PD) and erectile dysfunction^7–9^. It is a broad-spectrum dopamine agonist for all subtypes ranging from the dopamine D1 receptor to the D5 receptor. Apomorphine exerts protective effects by inhibiting oxytosis and stress-dependent non-apoptotic cell death via activation of the dopamine D4 receptor (D4R) in a micromolar order^10,11^.

Because oxytosis shares some concepts with ferroptosis proposed in recent years, one hypothesis is that the ferroptotic action of apomorphine is related to dopamine receptors. Ferroptosis is a common form of regulated cell death that is distinguished from apoptosis and necroptosis and is a form of lipid- and iron-dependent cell death associated with GSH depletion^12–14^. The mechanism of ferroptosis includes oxidative damage to cellular structures and dysfunction in membrane stability triggered by polyunsaturated fatty acid peroxidation^13,15^. It can be induced by GSH depletion using chemicals such as BSO, cystine-glutamate transporter inhibition, or GPX4 inhibition^12,16^. Among them, RSL3, which can inactivate GPX4, is the most commonly used inducer of ferroptosis, leading to excessive lipid peroxidation, which causes cell death^16^. There are also several biomarkers of ferroptosis, including protein markers, such as PTGS2 (which encodes COX-2), lipid peroxidation, and ROS^17–20^. In recent years, ferroptosis has been widely investigated for its involvement in various diseases, including mitochondrial diseases, neurodegenerative diseases, liver toxicity, liver fibrosis, hemochromatosis, and cardiomyopathy^13,21–24^. Also ferroptosis is regulated by various cellular metabolic pathways, including redox homeostasis, mitochondrial activity, and iron metabolism^13,15^.

In the present study, we found that apomorphine inhibited ferroptosis in fibroblasts from patients with mitochondrial disease as well as in normal controls, and the cell-protective effect was not related to the agonistic action of the dopamine receptor.

## Results

### Induction of ferroptosis by GSH depletion by BSO and prevention of BSO-induced ferroptosis by apomorphine in LS patient fibroblasts

**Figure 1:**
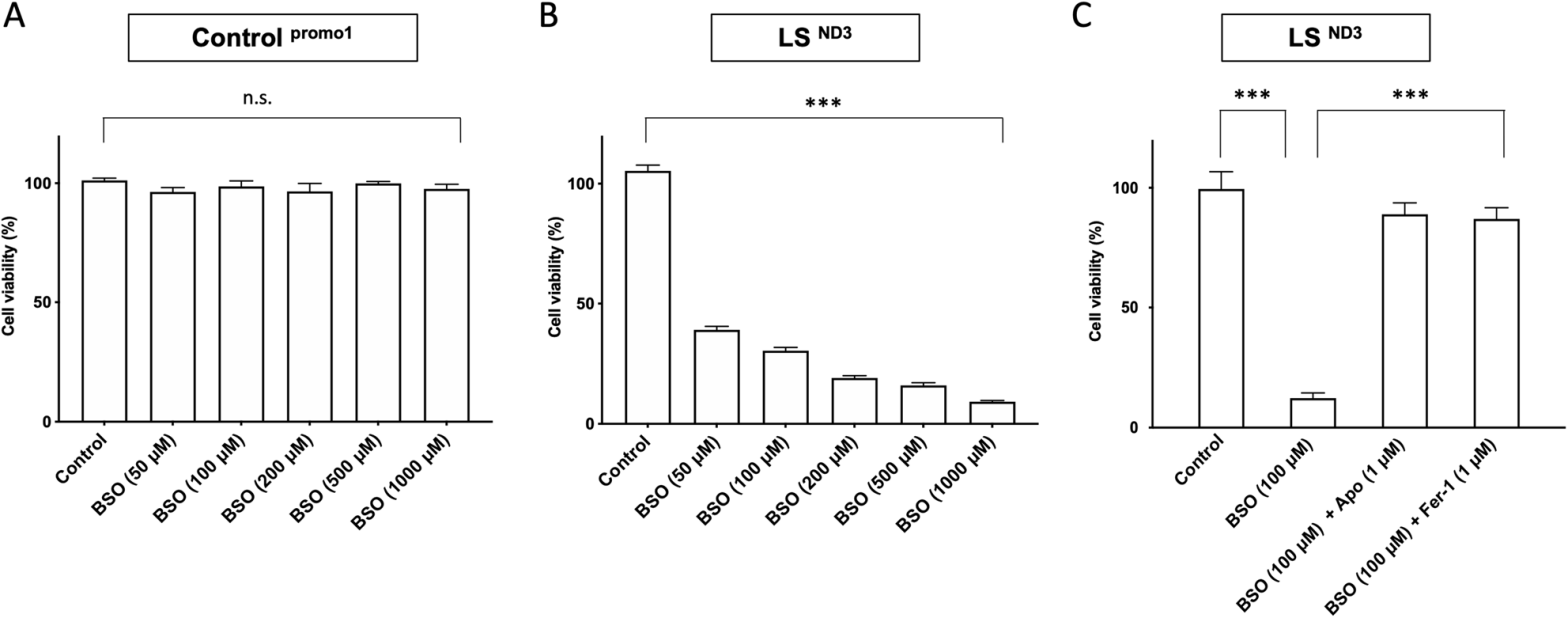
The cell viability assay for fibroblast cells from LS patients (LS^ND3^) under BSO-induced stress. While the fibroblasts of control subjects were able to maintain almost full viability (**A**), those from patients with LS showed enhanced BSO dose-dependent cell death (**B**). Under the conditions of 100 μM BSO treatment, we evaluated the effects of Fer-1 (1 μM), which is one of the most common ferroptosis inhibitors and Apo (1 μM). Fer-1 showed dramatic cell-protective effects, similar to those of Apo (**C**). *Apo* apomorphine, *Fer-1* Ferrostatin-1. Data (n = 6) are expressed as the mean ± SD ***P < 0.001. *n.s.* no statistical significance.

BSO reduced GSH production in a concentration-dependent manner^25^. Under these conditions, fibroblasts from LS patients showed enhanced cell death and were more vulnerable to BSO-induced cell stress than control fibroblasts, as previously described (Fig. 1A,B)^6^. We subsequently performed BSO-induced cell survival assays to investigate whether or not they were suppressed by adding ferrostatin-1 (Fer-1), one of the most common ferroptosis inhibitors^13,16^, to LS patient-derived fibroblasts. Fer-1 showed dramatic cell-protective effects under BSO-induced stress, and apomorphine showed similar effects (Fig. 1C).

### Protective effect of apomorphine against ferroptosis induced by BSO and RSL3

To evaluate the effects of apomorphine against ferroptosis, we examined the survival of LS fibroblasts in the presence of RSL3 as a common assay for ferroptosis screening, in addition to a BSO assay^16,26^. Prior to that, we examined the cell survival rate of control and patient fibroblasts under RSL3-induced stress, an inducer of ferroptosis. Fibroblasts obtained from LS, MELAS, mitochondrial cardiomyopathy, and KSS patients showed enhanced cell death in response to RSL3-induced stress, but this effect was less pronounced than the response to BSO-induced stress (Supplementary Fig. 1A–F).

**Figure 2:**
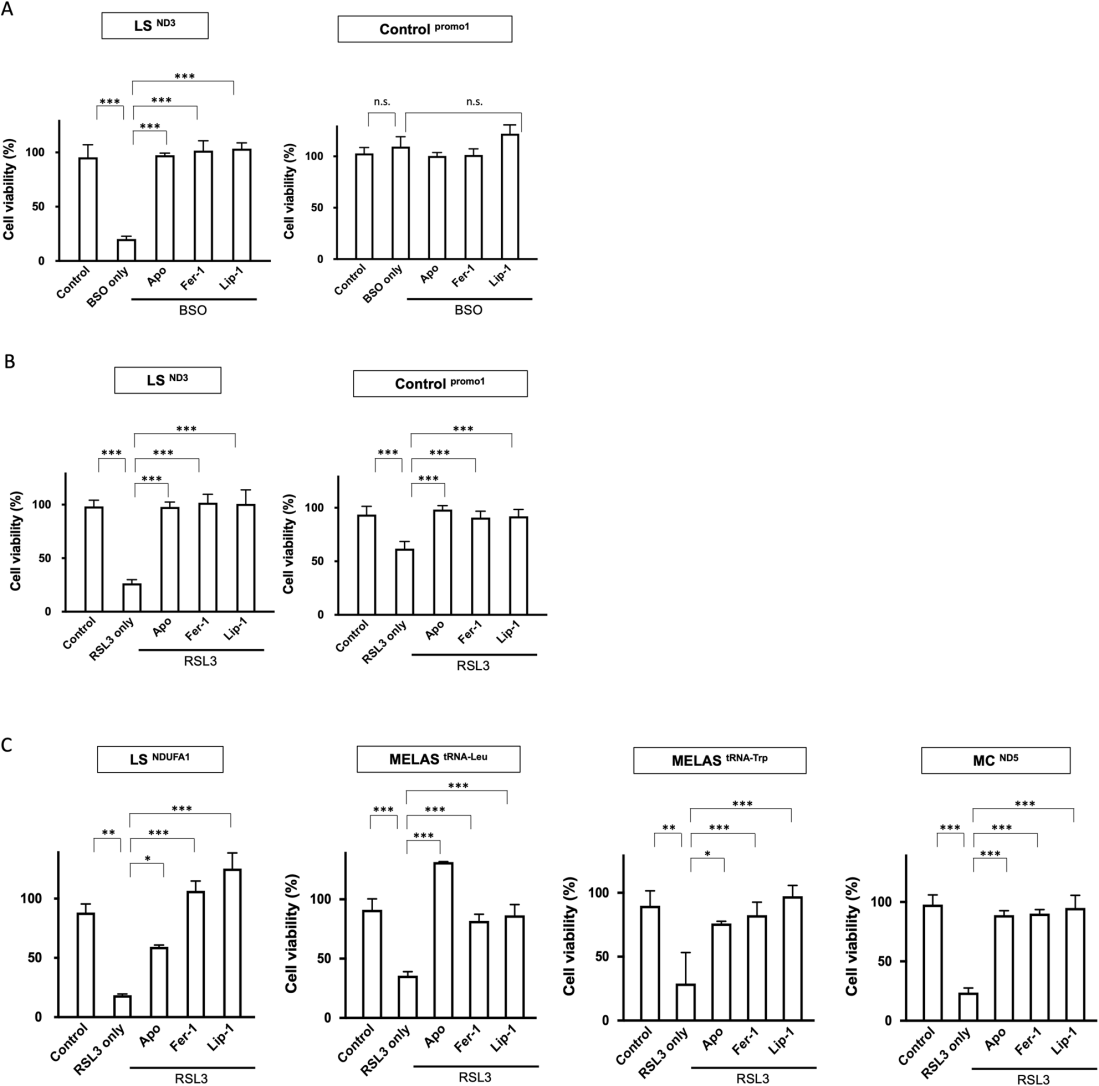
The anti-ferroptosis effect of apomorphine. (**A,B**) The cell viability assay of fibroblasts from LS patients (LS^ND3^) (left panel) and control (right panel) in the presence of 100 μM BSO and RSL3. (**C**) The cell viability assay of fibroblasts from patients with LS (LS^NDUFA1^), MELAS (MELAS^tRNA-Leu^, MELAS^tRNA-Trp^), mitochondrial cardiomyopathy (MC^ND5^), and KSS (KSS^large deletion^). The cell-protective effects of apomorphine were also shown in comparison to Fer-1, and Lip-1 in the presence of RSL3. *Apo* apomorphine, *Fer-1* Ferrostatin-1. Data (n = 6) are expressed as the mean ± SD *P < 0.05, **P < 0.01, ***P < 0.001. n.s. indicates no significance.

Subsequently, we compared the cell protective effect of apomorphine with that of Fer-1 and Liproxstatin-1 (Lip-1), well-known specific ferroptosis inhibitors^16,27,28^. Apomorphine in LS fibroblasts under ferroptosis induced by BSO showed cell-protective effects, but we could not investigate the effects of apomorphine in response to BSO-induced stress in control fibroblasts because BSO did not induce cell death in control fibroblasts (Fig. 2A). In the case of RSL3-induced stress, the difference in the survival between control and LS fibroblasts was less than that in response to RSL3-induced stress; apomorphine showed cell-protective effects in both control and LS fibroblasts. The effects of apomorphine were similar to those of Fer-1 and Lip-1 (Fig. 2B). We also examined other fibroblasts from different subtypes of mitochondrial diseases in the presence of RSL3 and obtained similar results (Fig. 2C).

**Figure 3:**
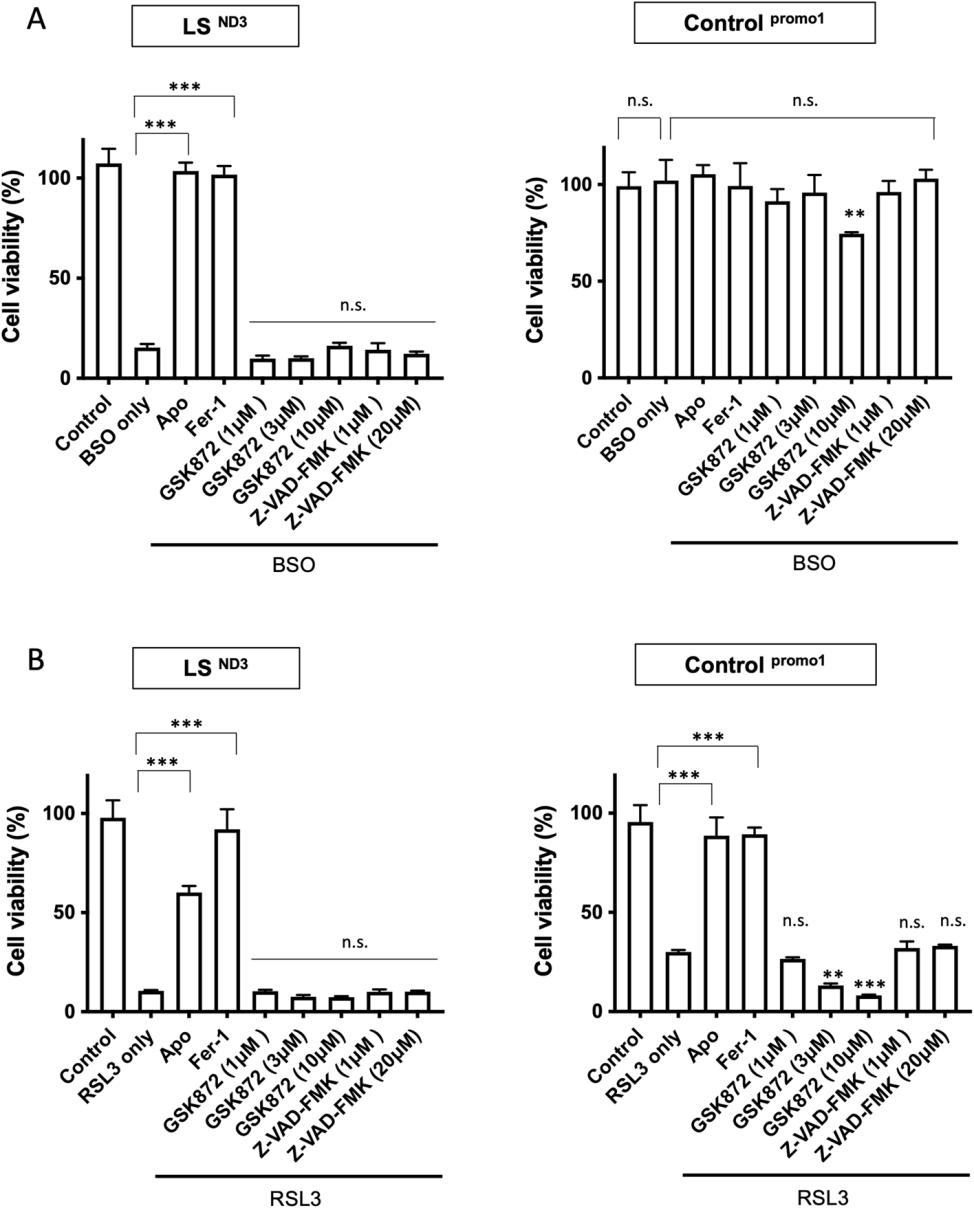
Cell viabilities by apoptosis and necroptosis inhibitors. The effects of an apoptosis inhibitor (GSK872) and a necroptosis inhibitor (Z-VAD-FMK) under conditions of 100 μM BSO- (**A**), and RSL3- (**B**) induced stress for fibroblast cells from LS patients (LS^ND3^) (left) and controls (right). *Apo* apomorphine, *Fer-1* Ferrostatin-1. Data (n = 3) are expressed as the mean ± SD *P < 0.05, ** P < 0.01, ***P < 0.001. n.s. indicates no significance.

We next examined the effects of various inhibitors of cell death pathways other than ferroptosis to rule out the possibility that apomorphine protects cells via other cell death pathways using GSK-872 (necroptosis inhibitor) or Z-VAD-FMK (apoptosis inhibitor). Although we could not examine the effects of these inhibitors in response to BSO-induced stress in control fibroblasts, BSO did not induce cell death in control fibroblasts, and neither GSK-872 nor Z-VAD-FMK showed any cell-protective effects against cell death induced by BSO and RSL3 (Fig. 3A,B).

These findings suggest that apomorphine protects against cell death by ferroptosis, but not by necroptosis or apoptosis, in control fibroblasts and fibroblasts derived from patients with mitochondrial disease.

### Measurement of intracellular ferroptotic metabolites in our cell lines by a BSO cell lipid oxidation assay

**Figure 4:**
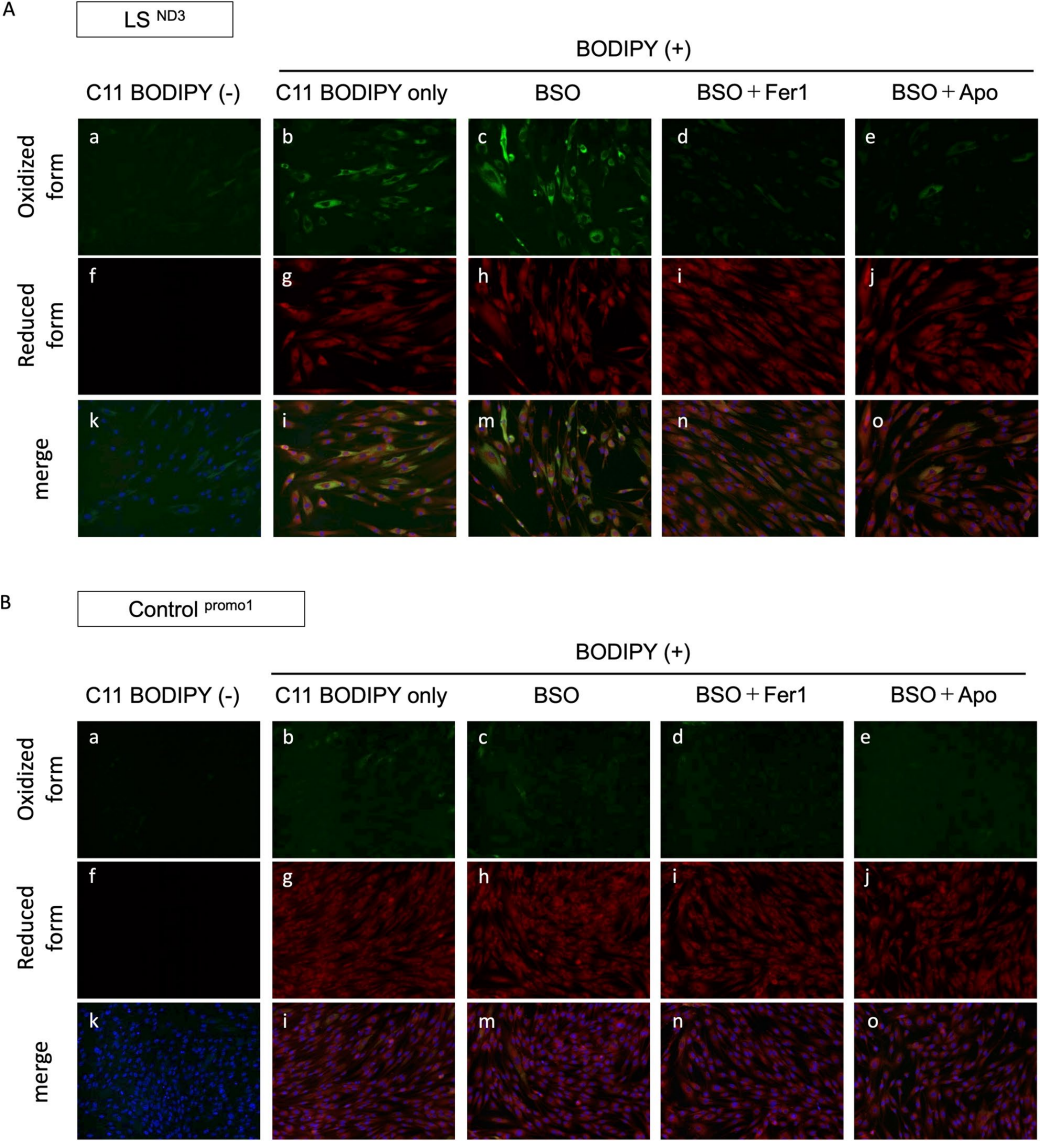
Results of a C11 BODIPY581/591 assay in fibroblasts from patients and controls. Measurement of intracellular ferroptotic metabolites by the BSO cell lipid oxidation assay. (**A**) Fibroblasts from LS patients (LS^ND3^) were treated with BSO (100 μM) for 24 h in the presence or absence of Apo (1 μM) and Fer-1 (1 μM). (**B**) Control fibroblasts were treated with BSO (100 μM) for 24 h in the presence or absence of Apo (1 μM) and Fer-1 (1 μM). Lipid peroxidation was assessed by C11 BODIPY581/591 staining. Representative images of C11 BODIPY581/591 staining in LS^ND3^ cells and control cells; Oxidized form (**a–e**), Reduced form (**f–j**), and merge (**k–o**). *Apo* apomorphine, *Fer-1* Ferrostatin-1.

Because the accumulation of lipid ROS is a hallmark of ferroptosis, we monitored the lipid ROS accumulation using C11 BODIPY 581/591 by confocal imaging (Fig. 4). The C11 BODIPY 581/591 changes the fluorescence from red to green upon oxidation^29^. Even in the absence of BSO, LS patient-derived fibroblasts showed the oxidized form of C11 BODIPY, which is indicative of lipid ROS (Fig. 4A_b). Upon BSO treatment, fibroblasts showed an increased lipid ROS accumulation (Fig. 4A_c). Furthermore, Fer-1, one of the most common inhibitors of ferroptosis, recovered the lipid ROS accumulation. When we examined the effect of apomorphine on the accumulation of lipid ROS in LS patient-derived fibroblasts treated with BSO, apomorphine decreased the lipid ROS accumulation and showed a similar effect to Fer-1 (Fig. 4A_d,e)^16^. In contrast, control fibroblasts showed almost no change in the degree of the lipid ROS accumulation (Fig. 4B_a–o).

### The assessment of the expression of genes related to ferroptosis in apomorphine-treated vs. untreated LS fibroblasts by real-time reverse transcription polymerase chain reaction (RT-PCR) and Western blotting

**Figure 5:**
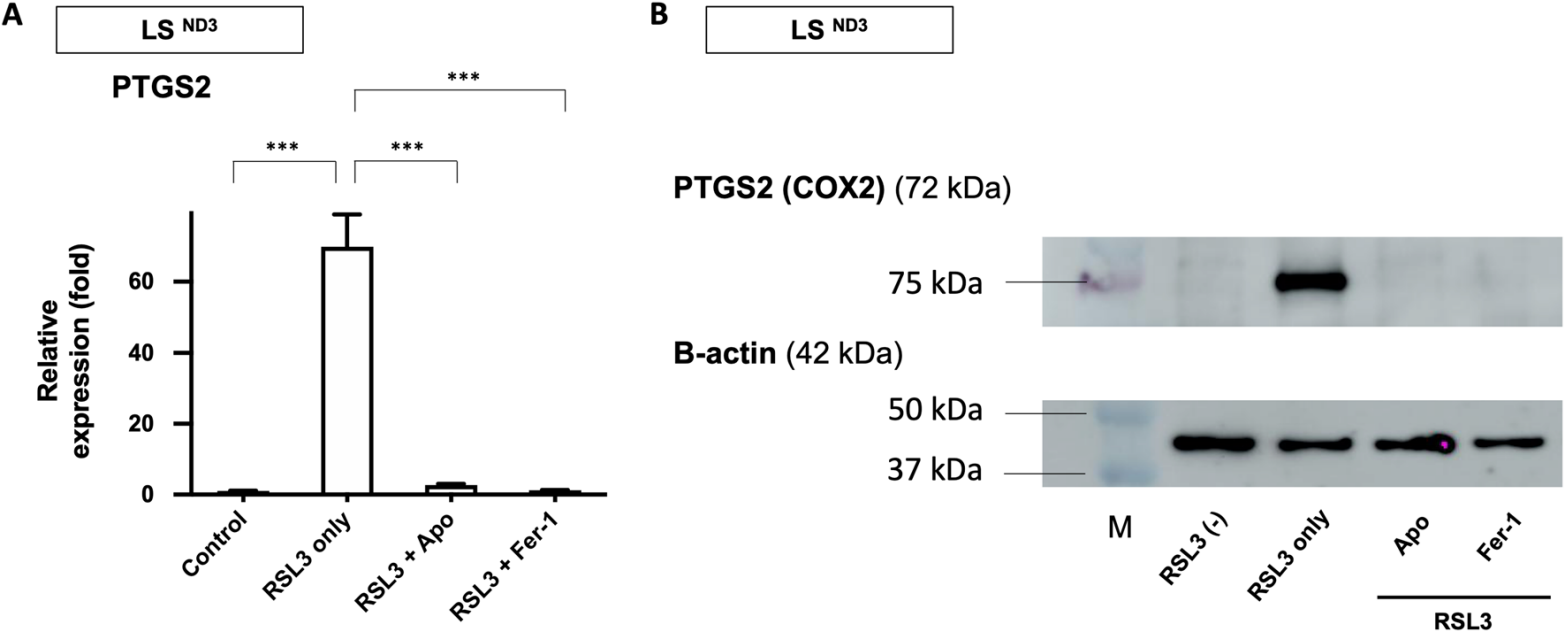
An expression analysis of prostaglandin-endoperoxide synthase 2 (PTGS2). (**A**) *PTGS2* mRNA levels in LS^ND3^ cells treated with RSL3 (100 nM) for 24 h in the presence or absence of Apo (1 μM) and Fer-1 (1 μM) were assessed using real-time RT-PCR. (**B**) PTGS2 protein levels in LS^ND3^ cells treated with RSL3 (100 nM) for 24 h in the presence or absence of Apo (1 μM) and Fer-1 (1 μM) were assessed by Western blotting. Full-length gels and blots are included in a Supplementary (Supplemental Fig. 3) as full-length dot blots. *Apo* apomorphine, *Fer-* Ferrostatin-1. Data are expressed as the mean ± SD; ***P < 0.001.

We assessed the expression of *prostaglandin endoperoxide synthase 2 (PTGS2)*, a biomarker of ferroptosis that encodes cyclooxygenase-2 (COX-2)^13,17–19^ in LS fibroblasts (LS^ND3^). Real-time RT-PCR showed that RSL3 upregulated *PTGS2* expression in LS fibroblasts and that this *PTGS2* upregulation was inhibited by apomorphine and Fer-1 treatment (Fig. 5A). Western blotting for PTGS2 showed that apomorphine significantly decreased the protein levels of PTGS2 in LS fibroblasts, and Fer-1 had a similar effect (Fig. 5B). We therefore demonstrated that apomorphine suppressed PTGS2, a key factor linking ferroptosis and inflammation, in LS fibroblasts.

### The analysis of the expression of genes related to ferroptosis in apomorphine-treated vs. untreated LS fibroblasts by real-time RT-PCR

**Figure 6:**
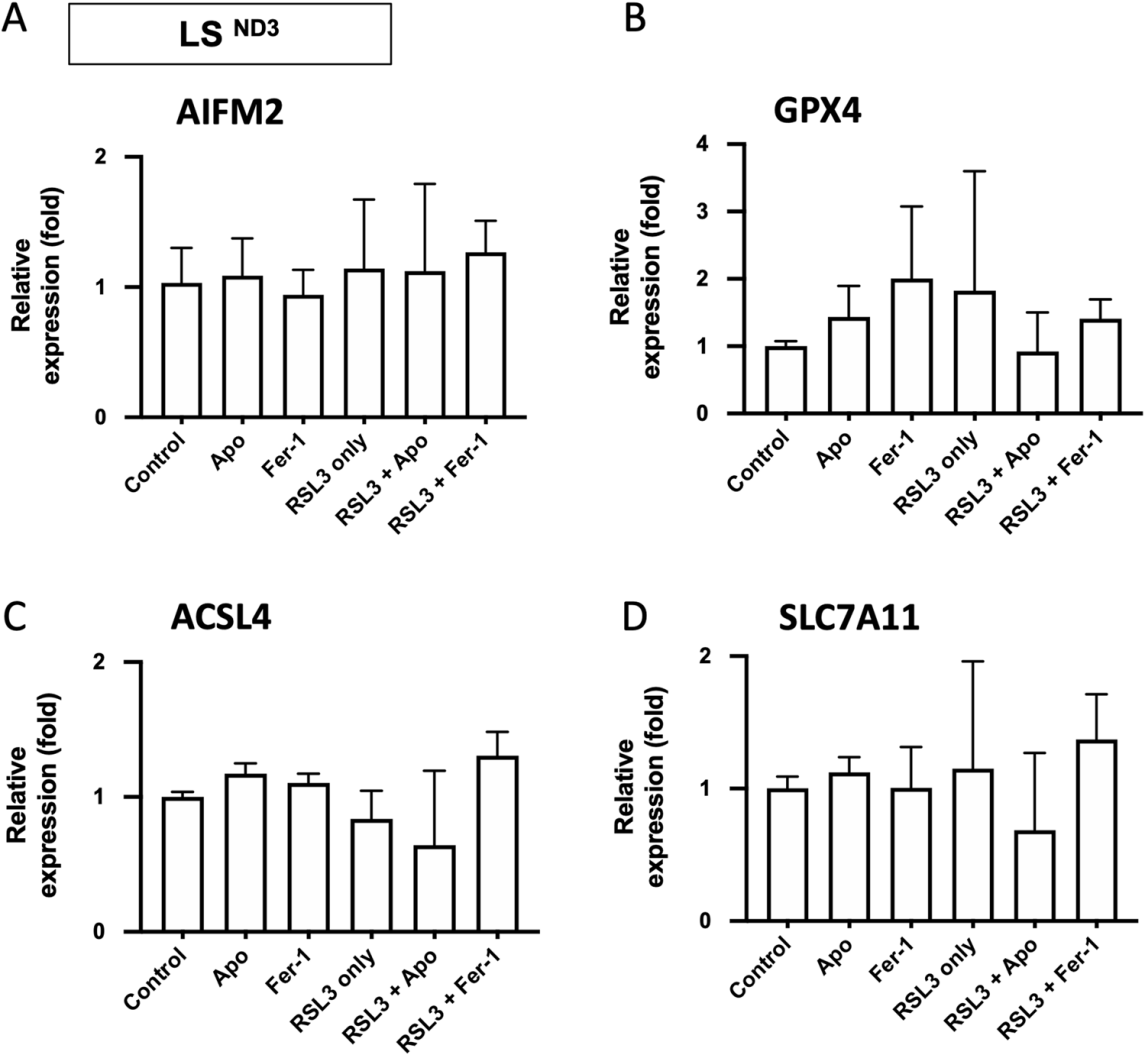
The mRNA levels of various ferroptosis marker genes. (**A**) *AIFM2* (encoding FSP1), (**B**) *GPX4,* (**C**) *ACSL4*, and (**D**) *SCL7A11* (Cystine/glutamate transporter) mRNA levels in LS^ND3^ cells treated with RSL3 (100 nM) for 24 h in the presence or absence of Apo (1 μM) and Fer-1 (1 μM) were assessed by real-time RT-PCR. *Apo* apomorphine, *Fer-1* Ferrostatin-1. Data are expressed as the mean ± SD *P < 0.05, ** P < 0.01, ***P < 0.001. n.s. indicates no significance.

To examine how apomorphine inhibits ferroptosis, we assessed the following key genes related to ferroptosis: *AIFM2* (encoding FSP1), *GPX4, ACSL4*, and *SLC7A11* (cystine/glutamate transporter)^13,17–19^. Real-time RT-PCR showed that apomorphine slightly suppressed the upregulation of *SLC7A11*; however, the differences were not statistically significant. Apomorphine also did not affect the mRNA expression of *AIFM2, GPX4,* or *ACSL4* (Fig. 6A–D).

### The anti-ferroptosis effect of apomorphine is not related to the dopamine receptor agonist action of apomorphine

Our previous screening did not show the cell protective effects of other dopamine agonists (Ropinirole hydrochloride and Pramipexole dihydrochloride; both specific to D2 receptors) (Supplemental Fig. 2). Therefore, to evaluate whether or not the agonistic action of apomorphine was related to its cell-protective effect, we examined the effect of the addition of agonists and/or antagonists of each dopamine receptor on the cell-protective effect of apomorphine.

**Figure 7:**
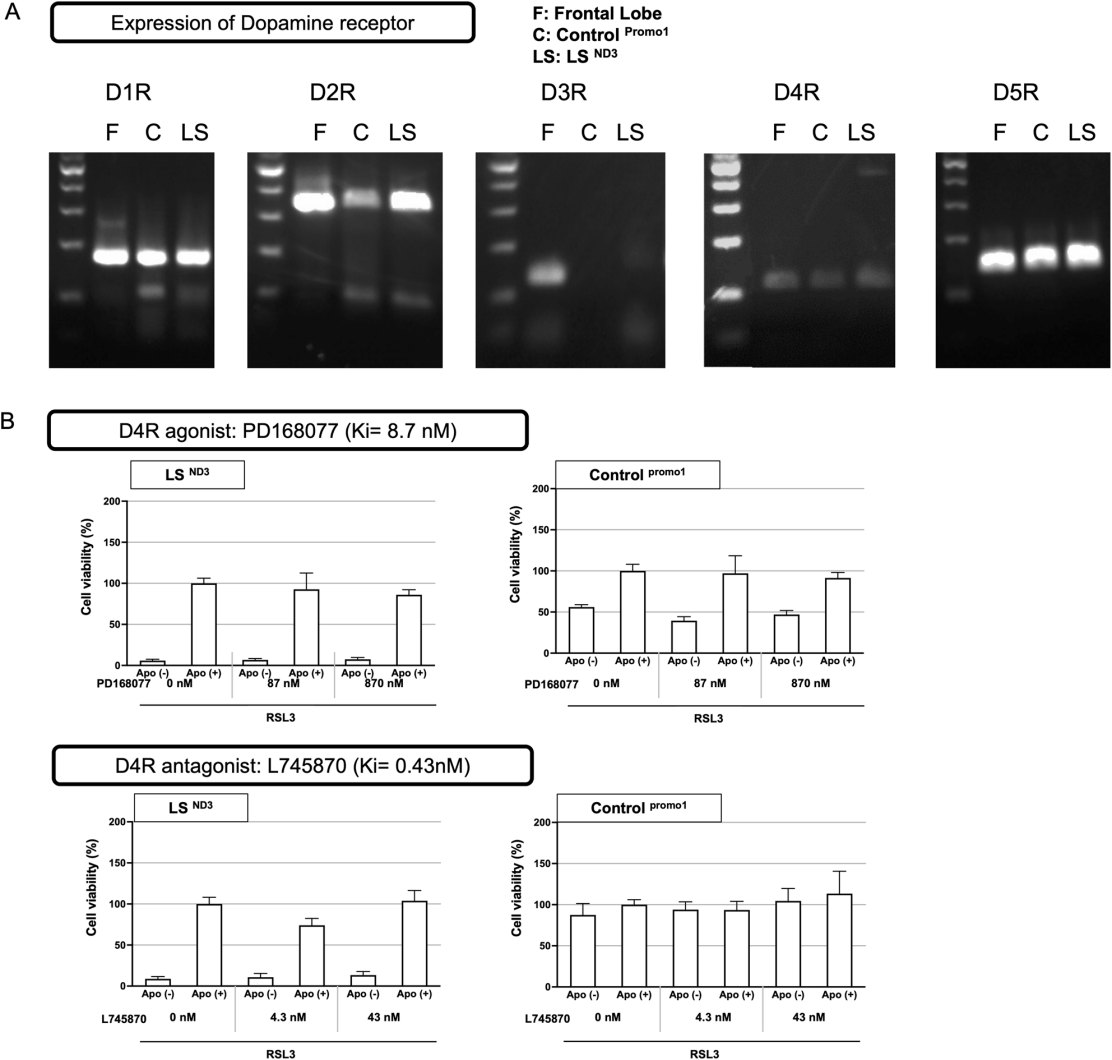

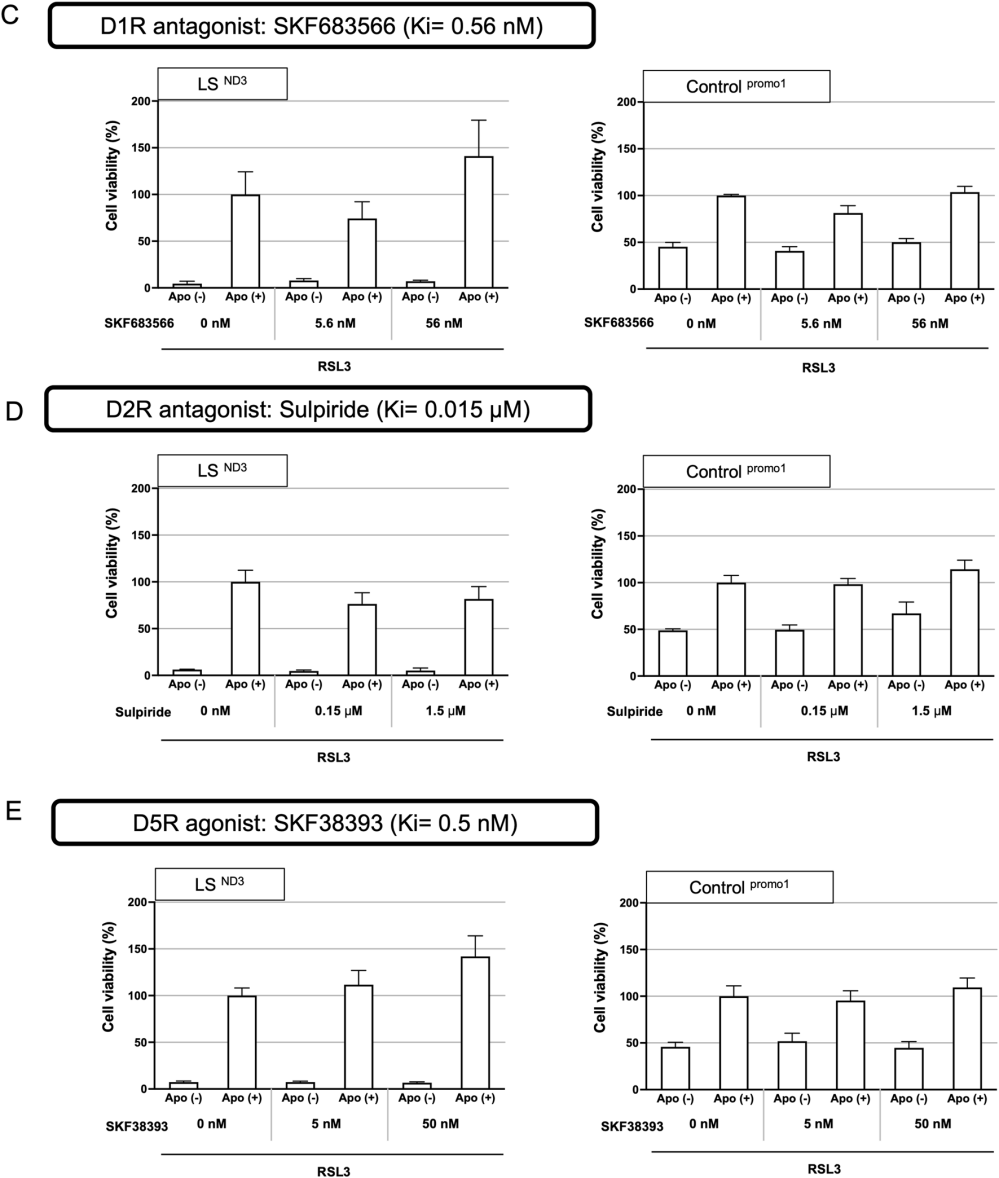
The expression of dopamine receptors (D1R-D5R) and interactions between apomorphine and dopamine receptor agonists or antagonists. (**A**) Agarose gel electrophoresis of the RT-PCR product of dopamine receptors. Results are shown from left to right in the order of D11R(size 184 bp), D2R (size 353 bp), D3R (size 137 bp), D4R (size 115 bp), and D5R (size 149 bp). In each column, the left lane shows the band of the frontal lobe as a positive control, the middle shows the band of control cells, and the right shows the band of LS cells. Original gels are presented in Supplementary Fig. 4. (**B–E**) The results of an RSL3-induced stressed cell viability assay to determine the influence of each dopamine receptor agonist or antagonist on the effects of apomorphine. The cell viability of the Apomorphine-treated group without RSL3 was used as 100% to compare other group’s cell viabilities (n = 3). (**B**) D4R agonist: PD168077 (Ki = 8.7 nM) and D4R antagonist: L745870 (Ki = 0.43 nM), (**C**) D1R antagonist: SKF683566 (Ki = 0.56 nM), (**D**) D2R antagonist: Sulpiride (Ki = 0.015 μM), (**E**) D5R agonist: SKF38393 (Ki = 0.5 nM).

Prior to this, the expression of each dopamine receptor in the cells was examined by RT-PCR. The expression of four types of dopamine receptors—D1R, D2R, D4R, and D5R—was confirmed in LS and control fibroblasts, but the expression of D3R was not detected in our fibroblasts (Fig. 7A). Therefore, we examined the action of each dopamine receptor on the protection of apomorphine, except for D3R.

First, we examined the contribution of D4R to the effects of apomorphine using agonists and antagonists of D4R, based on a previous report^10^. As shown in Fig. 7B, the D4R agonists did not enhance the effect of apomorphine, and the antagonists did not inhibit this effect. Similarly, antagonists of D1R and D2R did not affect the effect of apomorphine (Fig. 7C,D). Regarding D5R, as there was no commercially available antagonist for D5R, only the D5R agonist was used, but it did not provide any cell-protective effect (Fig. 7E).

## Discussion

We previously showed that apomorphine protects against BSO-induced stress with an EC50 of approximately 50 nM and improves the mitochondrial respiratory activity of fibroblasts from patients with LS and MELAS^6^. In the present study, we showed that fibroblasts from mitochondrial diseases including LS and MELAS were vulnerable to ferroptosis, and apomorphine markedly protected fibroblasts from ferroptosis induced by RSL3 in addition to BSO in various types of mitochondrial diseases as well as in normal fibroblasts. The effect of ferroptosis on cell protection was comparable to that of the specific ferroptosis inhibitors Fer-1 and Lip-1. We observed that BSO- or RSL3-induced ferroptosis was not inhibited by inhibitors of apoptosis (Z-VAD-FMK) or necroptosis (GSK-872), and *PTGS2,* which is known to be a marker of ferroptosis, was decreased by the addition of apomorphine. In addition, we confirmed that the effects of apomorphine were not related to its agonistic dopamine action.

Ferroptosis is an iron-dependent form of cell death caused by the accumulation of lipid hydroperoxides and is distinguished from other forms of cell death, such as apoptosis and necroptosis^14,16^. Ferroptosis occurs when GPX4 is directly or indirectly inhibited by GSH depletion, which leads to the accumulation of membrane lipid peroxidation and results in cell death^13,15,16^. RSL3 is commonly used as a specific inducer of ferroptosis and inactivates GPX4, leading to excessive lipid peroxidation, which causes cell death^16^. In contrast, ferroptosis can be suppressed by lipophilic antioxidants, inhibitors of lipid peroxidation, iron chelators, and polyunsaturated fatty acids^16^. Currently, several biomarkers of ferroptosis exist, including protein markers such as PTGS2, lipid peroxidation, and ROS^17–20^.

Apomorphine has been administered to patients with advanced PD as a rescue drug for “off” symptoms because of its short half-life. However, the continuous subcutaneous administration of apomorphine reportedly improves both motor symptoms as well as brain glucose metabolism and non-motor symptoms, such as the cognitive function, the executive function, and apathy in PD patients^30,31^. PD is known for the characteristic pathology of progressive dopaminergic neuronal death associated with iron accumulation, and it is suggested to be driven in part by ferroptosis^32,33^. The anti-ferroptosis effects of apomorphine shown in this study may explain these neuroprotective effects. To test this hypothesis, we need to study the effects in cells derived from patients with PD and animal models.

We have already shown that apomorphine suppresses the production of ROS^6^. In the present study, we showed that apomorphine prevented lipid peroxidation and upregulation of the mRNA expression of *PTGS2* (Fig. 5), which are key features of ferroptosis. Studies on cancer cells have indicated that ferroptosis can directly increase the expression of *PTGS2* and cause inflammation by accelerating AA metabolism and promoting the secretion of pro-inflammatory molecules^34,35^. The downregulation of *PTGS2* may be related to the inhibition of various cytokines and chemokines by apomorphine^6^. Recently, ferroptosis has been suggested to be involved in epilepsy with mitochondrial disease^12^. Our study also supports the hypothesis that fibroblasts from various mitochondrial diseases are vulnerable to ferroptosis.

Apomorphine reportedly protects against glutamate-induced oxidative cell death via dopamine receptors, especially D4, in the HT22 cell line^10^. In this assay system, the protective effect was reversed by D4 antagonists but not by D1 or D2 antagonists. A selective D4 agonist also protects neurons from glutamate-induced cell death. However, in our assay system using fibroblasts, the D4 agonist did not exert cell protective effects, and the D4 antagonist did not prevent the cell-protective effect of apomorphine. Furthermore, our data do not support the involvement of subtypes D1, D2, and D5. Therefore, we conclude that the cell-protective effect of apomorphine is not related to dopamine receptors in our system. Dopamine agonistic action causes several common side effects, especially digestive symptoms, such as nausea, vomiting, loss of appetite, and constipation^36^. Our research presents a promising avenue for the potential development of apomorphine derivatives without dopamine agonist activity, which offer cellular protection without the undesirable effects associated with dopamine agonists.

The limitation of the present study was that we were unable to identify the mechanism underlying the protection from ferroptosis, as we only performed an RT-PCR assay of key inhibitory genes for ferroptosis. Elucidation of the binding proteins that explain anti-ferroptosis and their downstream signaling cascades is the next research question that should be explored.

In conclusion, we found that fibroblasts from patients with mitochondrial diseases were vulnerable to ferroptosis, which is inhibited by apomorphine in this study. The cell-protective effect of apomorphine has long been believed to be a result of D4R agonistic action, but we first revealed that at least part of its anti-ferroptosis effect is not related to the dopamine receptor agonist action. Our study suggests that ferroptosis may be a potential therapeutic target for mitochondrial disease and also provides hope for the creation of new drugs that maintain their cell-protective effect without the dopamine agonist effect, to avoid the adverse effects of dopamine.

## Materials and methods

### Subjects

This study was approved by the Jichi Medical University Clinical Research Ethics Committee (Approval Number: J21-014) and all methods of this study were performed in accordance with the relevant guidelines and regulations. Fibroblasts were obtained from six patients at Jichi Medical University, Kanagawa Children’s Medical Center, and Saitama Medical University. The collection was conducted under the approval of the Jichi Medical University Clinical Research Ethics Committee, the Kanagawa Children’s Medical Center Review Board and Ethics Committee, and the Ethics Committee of Saitama Medical Center, Saitama Medical University, with approval numbers J21-014, H2021-094, and 11303-06, respectively. Written informed consent was obtained from the parents of each patient. And we used fibroblasts from healthy individuals as controls (normal human dermal fibroblasts purchased from PromoCell Company [#C-12300; Heidelberg, Germany]). Our four patients were diagnosed with LS and MELAS, as previously described^6^. Two of the patients had genetically identified cases of LS, including one with an m.10158 T>C, p(S34P) mutation in *MT-ND3* (Case 1; LS^ND3^) and one with a c.55 C>T, p(P19S) mutation in *NDUFA1* (Case 2; LS^NDUFA1^)^37,38^. Both ND3 and NDUFA1 are subunits of Complex I in the mitochondrial respiratory chain. Fibroblasts were also obtained from two patients with MELAS, including one with an m.3243 A > G mutation in tRNA-Leu (Case 3; MELAS^tRNA-Leu^) and one with an m.5541 C > T mutation in tRNA-Trp (Case 4; MELAS^tRNA-Trp^)^39^. We additionally obtained fibroblasts from patients with two other types of mitochondrial diseases: mitochondrial cardiomyopathy (Case 5; MC^ND5^) and Kearns-Sayer syndrome (KSS) (Case 6; KSS^large deletion^). The patient with mitochondrial cardiomyopathy who presented with HCM and died at 4 months old had an m.13513G > A mutation in the ND5 subunit of complex I (*MT-ND5* m.13513G > A), with 78.87% heteroplasmy. The patient with KSS was genetically identified as having a large deletion (m.8290-13802) of mitochondrial DNA (Table 1). Fibroblasts with fewer than 20 passages from patients and controls were used in the experiments. The heteroplasmic rate was analyzed by deep sequencing of mutated regions.

**Table 1:** Fibroblast cell lines from patients with mitochondrial disease.

Case	Cell ID	Disease	Age (years)	Gene mutation	Protein	Mutation rate (%)
1	LS^ND3^	Leigh	0	m.10158 T > C, p.(S34P)	ND3	90
2	LS^NDUFA1^	Leigh	5	c.55 C > T, p.(P19S)	NDUFA1	Nuclear gene
3	MELAS^tRNA-Leu^	MELAS	14	m.3243 A > G	(tRNA-Leu)	21
4	MELAS^tRNA-Trp^	MELAS	23	m.5541 C > T	(tRNA-Trp)	49
5	MC^ND5^	mitochondrial cardiomyopathy	0	m.13513 G > A p.(D393N)	ND5	79
6	KSS^large deletion^	KSS	1	m.8290-13802del	–	–
	Promo1	Control	0	–	–	–

Respiratory chain activities from fibroblasts: LS^ND3^—Complex I 9.8%, II 93.9%, III 94.5%, IV 47.6%, and CS 100.9%. LS^NDUFA1^—Complex I 27.6%, II 104.3%, III 70.1%, IV 77.7%, CS 77.4%. MELAS^tRNA-Leu^—Not available. MELAS^tRNA-Trp^—Complex I 48%, II 103%, III 65%, IV 18%, MC^ND5^—not available. KSS^large deletion^—not available.

*LS* Leigh syndrome, *MELAS* myopathy encephalopathy, lactic acidosis, and stroke-like episodes, *MC* mitochondrial cardiomyopathy, *KSS* Kearns-Sayer syndrome.

### Cell culture and growth conditions

Fibroblasts from patients were cultured in 1.0 g/L low-glucose Dulbecco’s Modified Eagle’s medium (DMEM) supplemented with 10% fetal bovine serum (FBS), 100 units/mL penicillin, and 100 μg/mL streptomycin. Cells were incubated at 37 °C in 5% CO_2_.

### Reagents

l-Butionine (S, R)-sulfoximine (BSO, No. B690270), a glutathione synthesis inhibitor, was purchased from Wako Pure Chemical Industries (Tokyo, Japan). RSL3 (No. S8155) was purchased from Selleck Chemicals (Houston, TX, USA). Fer-1 (No. SML0583) and Lip-1 (No. SML1414) were obtained from Sigma-Aldrich (St. Louis, MO, USA). Z-VAD-FMK (No. 3188-v) and GSK-872 (No. HY-101872) were purchased from Peptide Institute Inc. (Osaka, Japan) and MedChemExpress (Shanghai, China), respectively. All reagents were dissolved in dimethyl sulfoxide (DMSO).

### Cell viability assays

The BSO assay was performed as previously described^6^. We performed several cell viability assays to investigate ferroptosis. Ferroptotic cell death was induced in fibroblasts by ferroptosis inducers (RSL3 at 50–100 nM). In these cell viability experiments, fibroblasts were cultured to semi-confluence and plated at 5,000 cells per well in a 96-well culture plate in normal medium. After incubation for 24 h, apomorphine and several compounds were added as positive controls (Fer-1, or Lip-1) and cultured in the assay medium. All compounds were applied at a concentration of 1 μM unless otherwise indicated. After incubating the cell plates for 24 h at 37 °C (95% humidity and 5% CO_2_), a cell viability assay was performed using Cell Count Reagent SF (Nacalai Tesque, Kyoto, Japan). Fluorescence intensity was measured using a Benchmark Plus microplate reader (Bio-Rad, Hercules, CA, USA) according to the manufacturer’s instructions.

### The assessment of lipid peroxidation

Lipid peroxidation was examined using the fluorescent dye C11-BODIPY581/591 (No. D3861; Thermo Fisher Scientific, Waltham, MA, USA)^29^. Fibroblasts were cultured to semi-confluence and then plated at 5,000 cells per well in a 96-well culture plate in normal medium. After incubation for 24 h, BSO and several other compounds (e.g. apomorphine and Fer-1) were added to each well. After incubation for 24 h, the cells were labeled with 5 μM C11-BODIPY581/591 for 30 min in the assay medium. The nuclei were stained with Hoechst 33,342. Representative images were obtained using a Keyence All-in-One Fluorescence BZ-X810 microscope (Keyence Co., Itasca, IL, USA).

### RNA extraction and real-time RT-PCR

Total RNA was extracted from fibroblasts using an RNeasy® Mini Kit (QIAGEN, Valencia, CA, USA) according to the manufacturer’s instructions^40^. Total RNA was reverse transcribed to cDNA, followed by amplification by PCR with a Superscript® VILO cDNA synthesis kit at 60 °C (Invitrogen; Thermo Fisher Scientific). Real-time RT-PCR was performed using the SYBR Green system with a primer set that amplified a fragment of the target genes to measure the mRNA expression. The following primers were used: *PTGS2* (Forward primer 5′-GCCTGAATGTGCCATAA-GACTGAC-3′, Reverse primer 5′-AAACCCACAGTG-CTTGACACACA-3′), *AIFM2* (Forward primer 5′-ATGGTTCGGCTGACCAAGAG-3′, Reverse primer 5′-GCCACCACATCATTGGCATC-3′), *GPX4* (Forward primer 5′-GCCTTCCCGTGTAACCAGT-3′, Reverse primer 5′-GCGAACTCTTTGATCT-CTTCG-3′), *ACSL4* (Forward primer 5′-CCCTGAAGGATTTGAGATTCACA-3′, Reverse primer 5′-CCTTAGGT-CGGCCAGTAGAAC-3′), *SLC7A11* (Forward primer 5′-ATGCAGTGGCAGTGA-CCTTT-3′, Reverse primer 5′-GGCAACAAAGATCG-GAACTG-3′), and *GAPDH* (Forward primer 5′-CTTTGTCAAGCTCATTTCCTGG-3′, Reverse primer 5′-TCTTC-CTCTTGTGCTCTTGC-3′). The reactions were performed in triplicate. Gene expression was normalized to that of *GAPDH*, and the data were analyzed in the Excel software program, version 16.75 (Microsoft, Redmond, WA, USA) using the ΔΔCt method.

### Western blotting

For whole-cell extracts, cells were lysed in TNE buffer (20 mM Tris–HCL at pH 7.4, 150 mM NaCl, 1 mM EDTA at pH7.4). Protein concentrations were determined using the Qubit® Protein Assay Kit (Invitrogen; Thermo Fisher Scientific). Whole-cell lysates were mixed with an equal volume of 2 × sodium dodecyl sulfate (SDS) sample buffer and boiled. Western blotting was performed using 20 μg of total protein, and immunoprecipitation was performed using XV PANTERA GEL (NXV-361HP; DRC Co., Ltd, Tokyo, Japan). Total cell proteins were separated by 10% SDS–polyacrylamide gel electrophoresis (PAGE) and transferred to a PVDF membrane by electrotransfer. The membrane was blocked for 1 h at room temperature (RT) using 5% skimmed milk/phosphate-buffered saline with Tween (PBST) and then incubated with the following primary antibodies: rabbit monoclonal anti-PTGS2 (Cox2(D5H5)XP #12282; Cell Signaling, Danvers, MA, USA) at 1:1000 and mouse monoclonal anti-beta-actin (A1978; Sigma-Aldrich) at 1:5000 in PBST overnight at 4 °C. After washing with PBST 3 times for 10 min each, the membrane was incubated with the following secondary antibodies: anti-rabbit IgG horseradish peroxidase (HRP; Cell Signaling) at 1:3000 and anti-mouse IgG HRP (Santa Cruz Biotechnology, Santa Cruz, CA, USA) at 1:2000 for 1 h at RT. After washing with PBST 3 times for 15 min each, the membrane was incubated with Hyper HRP Substrate (TAKARA BIO INC., Ohtsu, Japan) for 2 min. Finally, chemiluminescence from the membrane was imaged using Amersham Imager 680 (GE Healthcare UK Ltd., Little Chalfont, UK). Protein intensities were measured using the ImageJ software program (https://imagej.net/ij/) Relative protein levels or protein abundances were normalized to those in the control group.

### Detection of dopamine gene receptor (DRD1-DRD5) expression on control and patient-derived fibroblasts by RT-PCR

RNA was extracted from isolated control and LS patient fibroblasts (LS^ND3^) using an RNeasy Mini Kit (QIAGEN) according to the manufacturer’s protocol. Commercial human Adult Normal Tissue: Brain: Frontal Lobe (BioChain, Newark, CA, USA) was used as the Positive Control. Total RNA (1500–2000 ng) was reverse-transcribed into first-strand cDNA using the Superscript VILO cDNA Synthesis kit (Invitrogen; Thermo Fisher Scientific). RT-PCR was performed using intron-spanning primers (Supplemental Table 1). The D1R/D2R/D5R cycle conditions were as follows: 94 °C for 2 min for polymerase activation, followed by 45 cycles at 98 °C for 10 s, 62 °C for 30 s, 68 °C for 1 min. The D3R cycle conditions were as follows: 94 °C for 2 min for polymerase activation, followed by 45 cycles of 98 °C for 10 s, 62 °C for 15 s, 72 °C for 1 min. The D4R cycle conditions were as follows: 94 °C for 2 min for polymerase activation, followed by 5 cycles of 98 °C for 10 s, 68 °C for 1 min, followed by 5 cycles of 98 °C for 10 s, 66 °C for 1 min, followed by 5 cycles of 98 °C for 10 s, 64 °C for 1 min, followed by 25 cycles of 98 °C for 10 s, 62 °C for 1 min, 68 °C for 7 min. The products were resolved on a 2% agarose gel containing ethidium bromide and photographed.

### Interactions between apomorphine and dopamine receptor agonists or antagonists: Effects on cell viability

Cell viability assays to examine the influence of dopamine receptor agonists or antagonists on the effects of apomorphine were performed under RSL-induced oxidative stress. SKF 83566 cells (no. HY-103430A; MedChemExpress) was used as the D1R antagonist^41^, sulpiride (No. S4655; Selleck Chemicals) as the D2R antagonist^42^, PD168077 (No. HY-21098A; MedChemExpress) as the D4R agonist^43^; L745870 (No. HY-14325; MedChemExpress) as the D4R antagonist^44^, and SKF38393 (No. S7993; Selleck Chemicals) as the D5R agonist^45^. The concentrations used were determined based on the EC50 and Ki values^41–45^.

A cell viability assay was performed as previously described^6^. In brief, fibroblasts were cultured in 1.0 g/L low-glucose DMEM with 10% FBS at 37 °C in 5% CO_2_ until they reached semi-confluence. Fibroblasts were seeded at 5,000 cells per well in a 96-well plate. After 24 h of incubation, we divided the cells into 3 groups: a DR agonist- or antagonist-treated group, a DR agonist- or antagonist-treated group at a concentration 10 times the EC50 or Ki values, and a DR agonist- or antagonist-treated group at a concentration 100 times the EC50 or Ki values. Apomorphine was added at a final concentration of 1 μM. After 24 h of incubation, the cell survival rate was checked using Cell Count Reagent SF (Nacalai Tesque). The cell viability of Apomorphin-treated group without RSL3 was used as 100% to compare other group’s cell viabilities.

### Statistical analyses

The results are expressed as the mean ± standard deviation. Comparisons between multiple-group means were performed using a one-way analysis of variance with Bonferroni’s post-hoc test. Statistical significance was set at P < 0.05. Statistical analyses were performed using the GraphPad Prism software program version 9.3.1 (GraphPad Software Inc., La Jolla, CA, USA).

### Supplementary Information

Supplementary Figure 1.

Supplementary Figure 2.

Supplementary Figure 3.

Supplementary Figure 4.

Supplementary Table 1.

## Data Availability

The datasets and raw data used and/or analyzed during the current study are available from the corresponding author on reasonable request.
