# Supplementary figures and images for: Apomorphine is a potent inhibitor of ferroptosis independent of dopaminergic receptors
Source: Sci Rep. 2024 Feb 27;14:4820. doi: 10.1038/s41598-024-55293-1 (PMC10899610; doi:10.1038/s41598-024-55293-1)

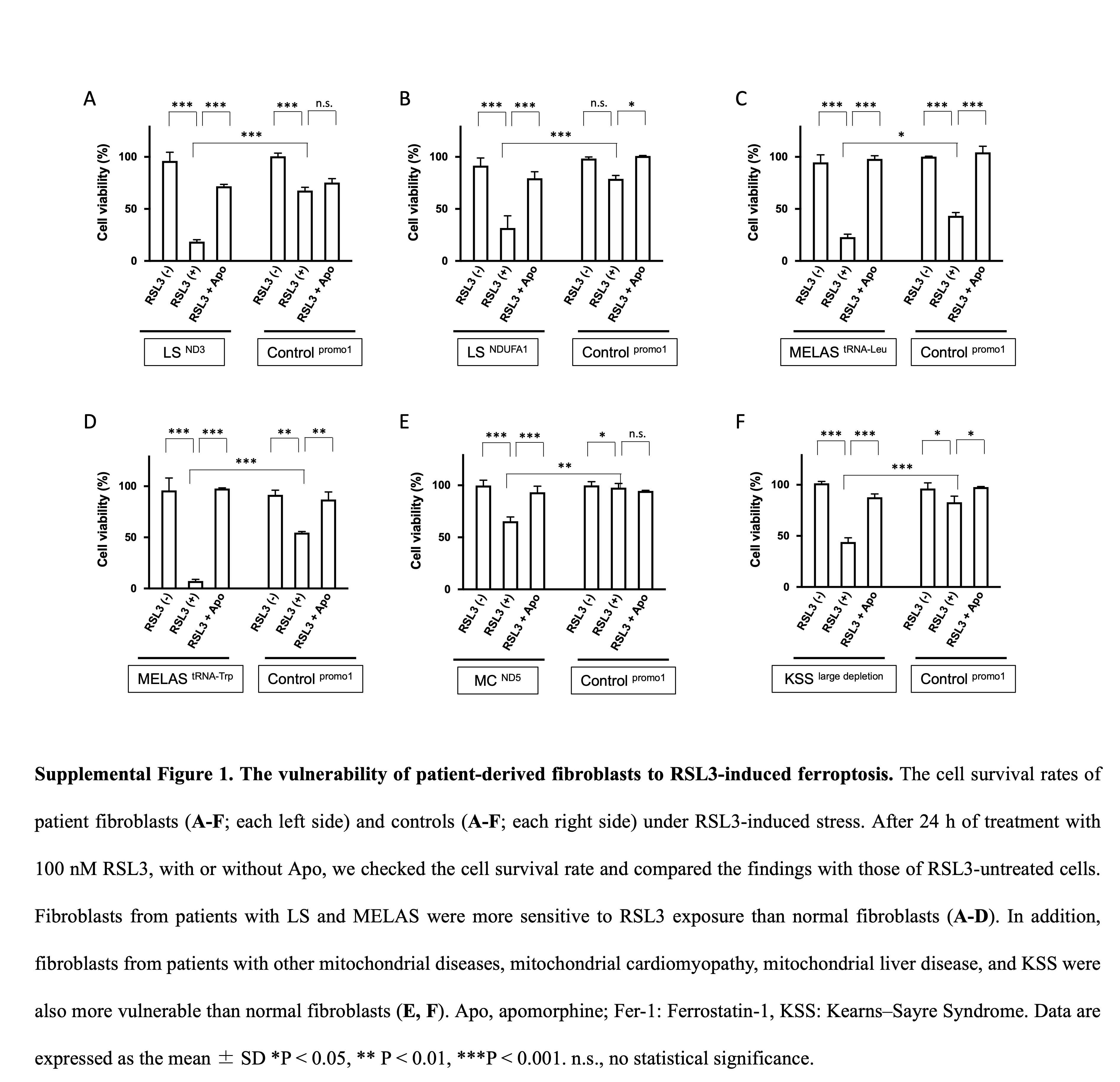

Supplement: Supplementary file 1 — Supplementary Figure 1. [file 41598_2024_55293_MOESM1_ESM.tiff]

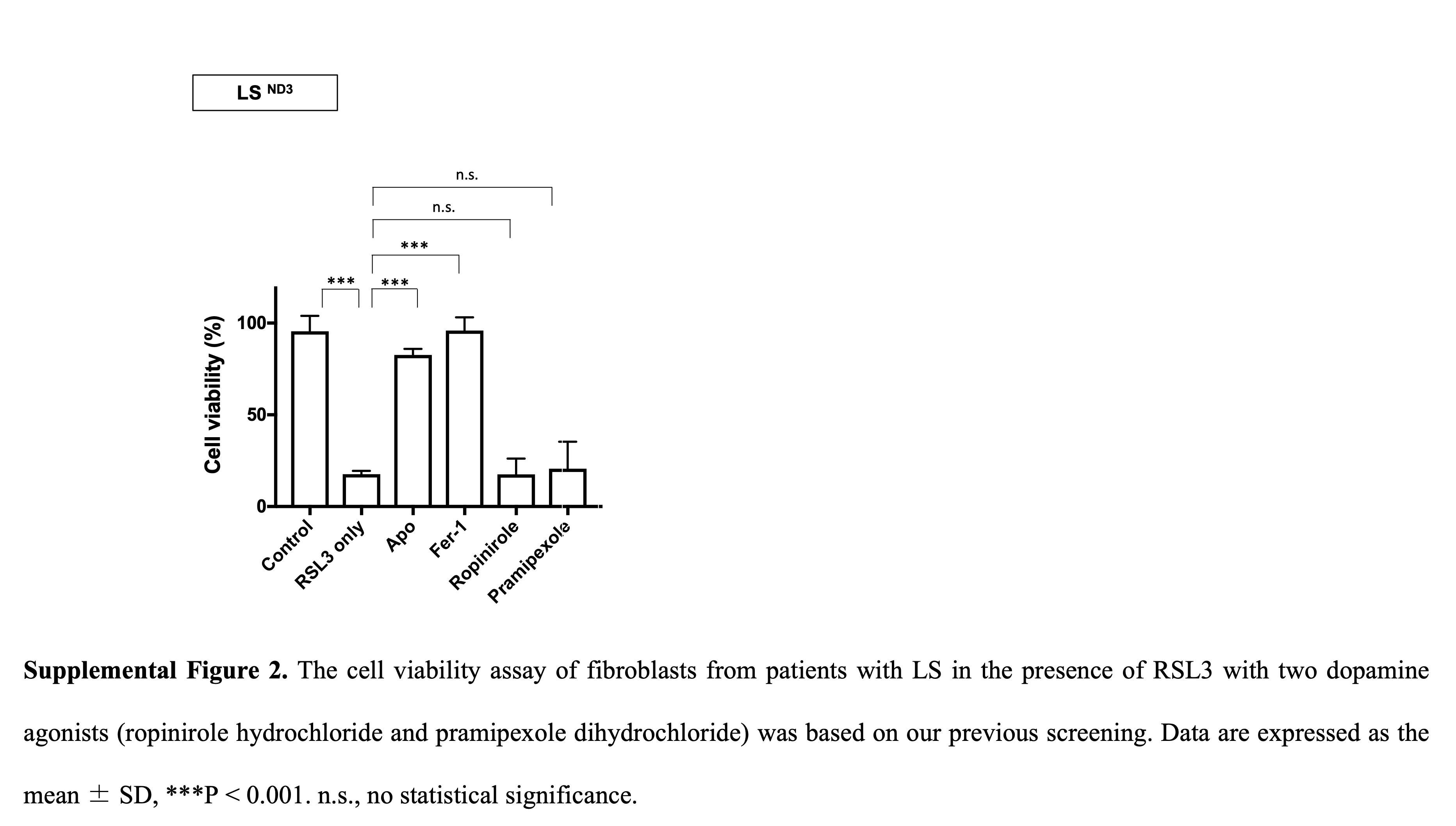

Supplement: Supplementary file 2 — Supplementary Figure 2. [file 41598_2024_55293_MOESM2_ESM.tiff]

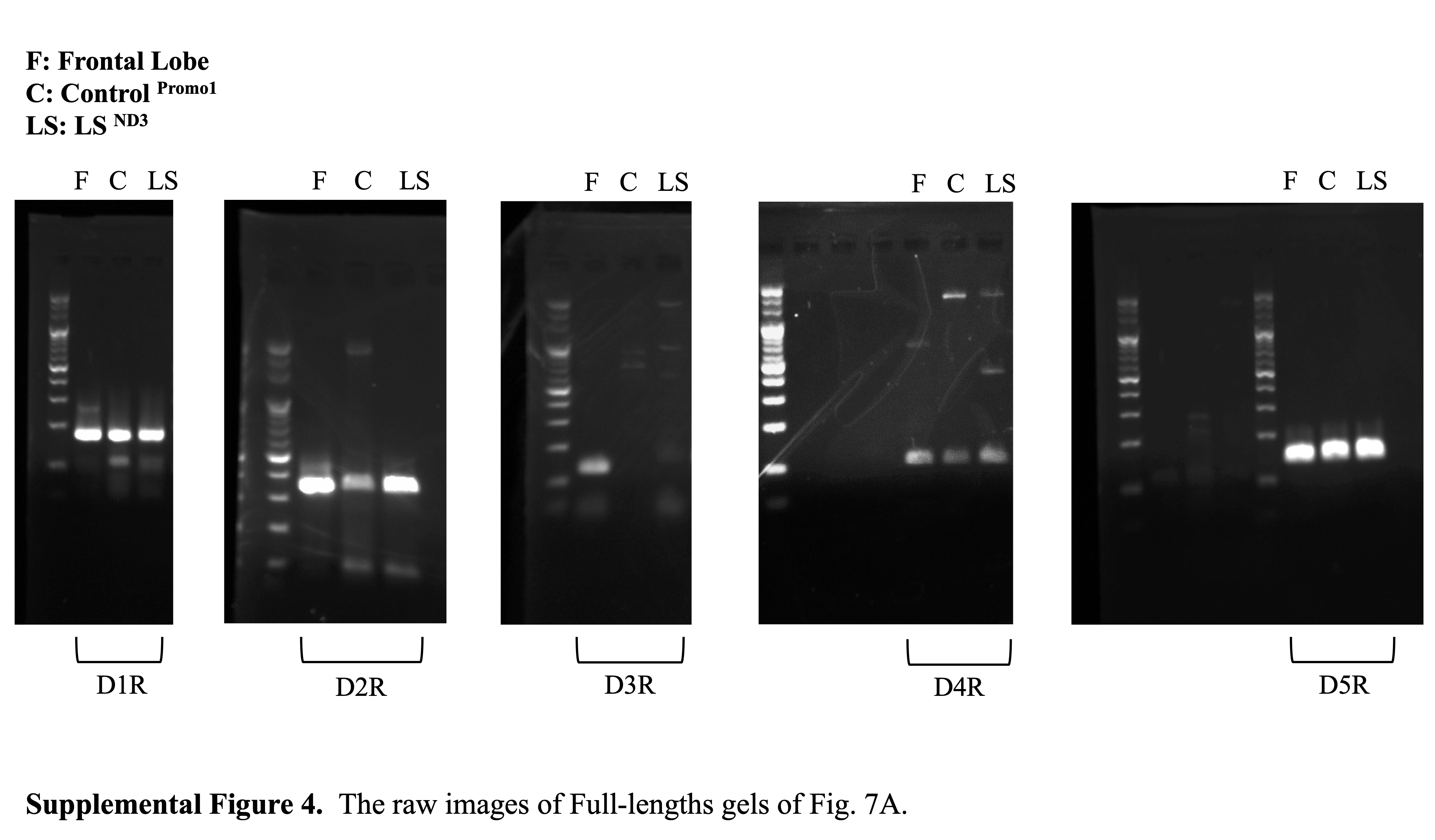

Supplement: Supplementary file 4 — Supplementary Figure 4. [file 41598_2024_55293_MOESM4_ESM.tiff]
